# Malnutrition contribution to the functional status and health related quality of life after COVID-19, a correlational follow-up study

**DOI:** 10.1038/s41598-024-65698-7

**Published:** 2024-07-01

**Authors:** Laura Alejandra Mejía Alonso, Eliseo Espinosa-Poblano, Sarahi de Regil López, Verónica Lemus Eslava, Jesús Guadalupe Serrano Sánchez, Carlos Paredes-Manjarrez, Andrés Tlacaelel Balderas-Chairéz, Juan Carlos Anda-Garay, José Adán Miguel-Puga, Kathrine Jáuregui-Renaud

**Affiliations:** 1 Unidad de Rehabilitación, Hospital de Especialidades del Centro Médico Nacional Siglo XXI, Instituto Mexicano del Seguro Social, Mexico city, Mexico; 2 Departamento de Inhaloterapia y Neumología, Hospital de Especialidades del Centro Médico Nacional Siglo XXI, Instituto Mexicano del Seguro Social, Mexico city, México; 3 Departamento de Nutrición y Dieta, Hospital de Especialidades del Centro Médico Nacional Siglo XXI, Instituto Mexicano del Seguro Social, Mexico city, México; 4 Departamento de Imagenología, Hospital de Especialidades del Centro Médico Nacional Siglo XXI, Instituto Mexicano del Seguro Social, Mexico city, México; 5 Departamento de Medicina Interna, Hospital de Especialidades del Centro Médico Nacional Siglo XXI, Instituto Mexicano del Seguro Social, Mexico city, México; 6 Unidad de Investigación Médica en Otoneurología, Instituto Mexicano del Seguro Social, Mexico city, México

**Keywords:** COVID-19, Malnutrition, Obesity, Functioning, Skeletal muscle, Diseases, Medical research, Signs and symptoms

## Abstract

To assess malnutrition contribution to the functional status and health related quality of life after hospitalization due to COVID-19 pneumonia, 66 selected adults referred for physical rehabilitation accepted to participate in the study; none of them required oxygen supply or had history of lung/musculoskeletal/neurological/immune/rheumatic disease or trauma, or contraindication for respiratory-function tests. At three evaluations, with 3 months in-between, assessments included: self-report of functional status, the St. George’s Respiratory Questionnaire, spirometry, the 6-min-walk-test, the MRC-scale, the 30-s sit-to-stand-test, the timed-up-and-go-test, nutritional status, and ultrasound imaging (vastus medialis and diaphragm). At referral, patients had nutritional deficits with protein deficiency, which gradually improved; while muscle thickness (of both vastus medialis and diaphragm) increased, along with muscle strength and mobility (ANOVA, *p* < 0.05). Contrarywise, the distance covered during the 6-min-walk-test decreased (ANOVA, *p* < 0.05), with a negative influence from excess body mass. During rehabilitation, health-related quality of life and functional status improved, with negative influence from a history of tobacco use and referral delay, respectively. After hospitalization due to COVID-19, early diagnosis of both protein deficiency and decrease of skeletal muscle thickness could be relevant for rehabilitation, while pondering the negative impact of excess body mass on submaximal exercise performance.

## Introduction

After acute COVID-19, patients may report persistent symptoms that have an impact on functioning^1^, and the use of health services^2,3^. A systematic review and meta-analysis of seven studies showed that 80% of 1951 adults reported at least one symptom, including fatigue, exercise intolerance, and dyspnoea^4^. Six months after hospital discharge, 25% of 1733 patients had reduced submaximal exercise capacity^5^ on the 6-min-walk-test (6MWT)^6,7^. A 2-years follow-up of 138 818 infected and 5 985 227noninfected individuals showed that COVID-19 sequelae contributed to 80.4 (95% Confidence Interval 71.6–89.6) and 642.8 (95%C.I. 596.9–689.3) disability-adjusted life years/1000 persons among non-hospitalized and hospitalized individuals, respectively^3^.

Evidence supports that the persistence of symptoms may be multifactorial, including inflammatory factors and deconditioning^8^; while malnutrition and reduced muscle mass contribute to functional limitations ^9–11^. Thirty days after hospital discharge, 47.2% of 288 patients had malnutrition^12^; four to 5 months from acute disease, a cohort study (n = 1230) showed a prevalence of malnutrition of 26% and 19% in non-hospitalized and hospitalized patients, respectively^13^.

After mild to moderate COVID-19, muscle biopsies have shown mitochondrial changes, inflammation, and capillary injury, among other abnormalities^14^. During the acute disease, muscle quantification allows mortality risk stratification of adult patients^15^. During recovery from the acute disease, 86% of 41 patients had quadriceps weakness^10^; three months after hospital discharge, 16% of 139 patients who required respiratory support^16^, and 58% of 34 patients referred to a Rehabilitation Unit^17^ had sarcopenia. Nevertheless, after acute COVID-19, patients may also have reduced diaphragm function^18–20^; though, sometimes it may not correlate with dyspnoea scores^20^.

During recovery from the acute disease, interaction among all these factors may affect functioning and quality of life. However, individualized follow-up is required to explore their potential contribution, while excluding comorbidities and treatment variability, and considering idiosyncrasies. The purpose of this study was to assess malnutrition contribution to the functional status and health related quality of life after hospitalization due to COVID-19 pneumonia, in adults who received a standardized treatment (in a single hospital) and were referred for physical rehabilitation. A follow-up was performed on the perceived level of functioning and health related quality of life, considering personal habits (alcohol/tobacco use), and assessing nutritional status, skeletal muscle thickness (vastus medialis and diaphragm) and strength, with performance evaluations, includeding mobility, submaximal exercise capacity and respiratory function.

## Methods

### Participants

After approval by the Research and Ethics Committees (R-2020–785-157), all procedures were performed in accordance with relevant guidelines and regulations, and with the declaration of Helsinki and its amendments. From July 16/2020 to May 4/2022, sixty-six consecutive patients appointed for rehabilitation exercises after hospitalization due to COVID-19 pneumonia, who fulfilled the selection criteria, gave their written informed consent to participate in the study.

The participants were 25 women and 41 men (25–85 years old, mean 51.3, standard deviation (S.D.) 14.7). All of them received institutional treatment^21^ in the same hospital; during hospital stay, 13 (19%) patients required intensive care. Due to personal reasons, two patients included in the analysis didn’t perform the second evaluation (n = 64); three more patients accepted to participate but were excluded because they performed just the first evaluation. The sample size was estimated to identify correlations ≥ 0.35 (n = 61 patients; two sided α = 0.05 and β = 0.02).

The selection of participants was performed according to their clinical records and preliminary medical evaluations, to confirm that none of them required oxygen supply or had history of previous lung/musculoskeletal/neurological/immune/rheumatic disease, or trauma, or contraindication for respiratory function tests^22^. During participation in the study, none of the patients received pharmacotherapy, other than that required for their individual comorbidities (mainly diabetes, high blood pressure).

### Procedures

After review of the clinical records, an inhouse questionnaire was administered to corroborate demographics and general health information, including alcohol/tobacco use. Then, evaluations were performed three times, **at **mean 139 (S.D. 72) days from discharge to rehabilitation referral for the first evaluation, 238 (71) days for the second evaluation, and 333 (79) days for the third evaluation. At each time point, by direct interview, patients were asked to report any persistent symptom interfering with their daily life activities, and to categorize their functional status as: 1. performing their daily life activities as usual or 2. with limitations or 3. inactive. Then, the following assessments were completed:

- *Nutritional assessment* was performed by experienced nutritionists, according to institutional procedures^23^, which comply with the Academy of Nutrition and Dietetic recommendations^24^ and the Global Consensus Around Core Diagnostic Criteria for Malnutrition in Adults in Clinical Settings^25^, and measurement of body height and mass, brachial circumference, and calf circumference. The nutritional diagnoses were classified as: 1. undernutrition, 2. adequate nutrition, 3. overweight (with or without protein deficiency), 4. obesity (with or without protein deficiency).
- *Ultrasound assessments* were performed by an experienced ultrasound specialist with the help of a single imaging resident. Images were stored as DICOM files (Enterprise-Imaging-Platform, Agfa-Health Care, Mortsel, Belgium), and they were evaluated off-line by two independent senior ultra-sound medical specialists, in the case of discordance, another specialist performed a third revision.
  *Vastus medialis.* Imaging was performed on the dominant leg (right leg on all subjects), in the supine position with a small cushion below the hollow of the knees; in the middle of the vastus medialis, at 50% of the distance from the central palpable point of the greater trochanter to the lateral condyle of the femur. Scans were performed using a linear transducer (Logiq E9, Logic 9 8.0 MHz; General Electric Healthcare, Waukesha, WI) after water-soluble gel was placed over the skin, with the transducer oriented parallel to the muscle fascicles and perpendicular to the skin. An appropriate transducer alignment was considered when several fascicles could be delineated without interruption across the image. After anatomical assessment, muscle thickness was measured as the distance between the deep and the superficial aponeurosis. Then, shear wave elastography was performed to assess muscle stiffness by the median propagation velocity among five measurements, with an interquartile range < 10%.
  *Diaphragm.* Imaging was performed in supine position (Logiq E9, General Electric Healthcare, Waukesha, WI). A convex transducer (Logic 9 3–5 MHz, General Electric Healthcare, Waukesha, WI) was placed over the right/left anterior subcostal region at the anterior axillary lines; it was directed medially, cranio- dorsally, so that the ultrasound beam reached perpendicularly the posterior third of the diaphragm. In B mode, the best view of the right/left hemidiaphragms were evaluated deep to the intercostal muscle layer and ribs, via the liver and the spleen windows respectively; adequate view of the left hemidiaphragm was not feasible in 5, 4 and 6 patients at each of the three evaluations. The diaphragm was observed as a structure composed by three layers, a non-echogenic central layer bordered by two hyperechogenic (peritoneal and pleural) layers. During quiet breathing, on M mode, the mobility was measured from the amplitude of its’ cranio-caudal maximum excursion; then, thickness was measured from the centre of the pleural line to the centre of the peritoneal line. An average of three measurements was estimated to give the thickness at the end of expiration and at the end of inspiration. A thickening fraction was estimated by dividing the average measurement at the end of inspiration minus the one obtained at the end of expiration, and then dividing the result by the average obtained at the end of expiration.
- *Muscle strength* was evaluated by an experienced medical doctor specialized in physical medicine and rehabilitation, including: 1. the Medical Research Council Scale^26^ that grades muscle strength in relation to the maximum expected for the muscle being explored (scale from 0 to 5), and 2. the 30-s sit-to-stand test^27^, in which the patient is asked to perform as many repetitions as possible in 30 s, after standing by a chair with feet pelvis-width apart, with hands crossed at shoulders, considering only those repetitions in which the patient touch the chair with the thighs or buttocks and return to the initial position by extending the knees and hips; with test–retest intraclass correlation > 0.80^27,28^, and correlation with leg-press performance > 0.70^27^.
- *Mobility assessment* was performed by the modified Timed-Up-and-Go test^29^, in which patients are instructed to sit on a chair, stand and walk a 3 m course at a rapid speed, to walk back to the chair and then to sit again; the time to the nearest tenth of a second is recorded from the command to “Go” to the time when the backside of the patient touch the chair, using a standard digital stopwatch.
- *Submaximal exercise capacity* was assessed by the 6MWT^6^, and spirometry^30^. Patients were asked to walk at their own pace along a 30 m long corridor, for 6 min. Before and after the test, assessments were performed on dyspnoea (Borg scale^31^, oxygen saturation (Advanced PO-100B, Miami FL), heart rate, respiratory rate, and blood pressure. The proportion of the predicted walking distance for each patient, was estimated as the actual distance covered during the test corrected by height, weight, age, and sex^32^, as follows: 218 (5.14 × height (cm)—532 × age (years) – (1.8 × weight (kg) + (51.31 × sex (1 for men and 0 for women)). Before the 6MWT, spirometry (EasyOne Air, ndd Medizintechnik AG, Zurich), Switzerland) was performed according to standard recommendations [30), to assess forced vital capacity (FVC), forced expiratory volume in the first second (FEV1), FEV1/FVC ratio, 25%, 50%, and 75% forced expiratory flow (FEF), and peak expiratory flow (PEF).
- *Respiratory Health related quality of life* was assessed by the self-administered St. George´s Respiratory Questionnaire^33^, which comprises three subscales: 1. symptoms (eight items), 2. activity (16 items), and 3. impacts (26 items). Scores are calculated by specific algorithms for each subscale, and the overall questionnaire scores range from zero = no impairment to 100 = maximum impairment; with a score coefficient of variation over 2 weeks of 19%^34^.

According to the institutional procedures, nutritional recommendations were provided^23^, and individual goals for rehabilitation exercises were set^35^ by the same experienced medical doctor specialized in physical medicine and rehabilitation. The aim of the physical therapy was to attain the highest level of independence and functional ability; training was tailored to individual Specific, Measurable, Achievable, Relevant, and Time-bound (SMART^36^) goals, in agreement with the FITT framework (frequency, intensity, time, and type)^37^, and with demonstrative videos and infographics for standardization. The program comprised 3 to 5 sessions/ week (30–60 min each), partitioned into 10–15 min periods, to include three moments: warm-up, workout, and recovery. Warm-up comprised slow-paced walking (3 to 5 min), followed by gentle stretching (3 min) targeting scapular and pelvic muscle groups. Workout encompassed multimodal exercises, including aerobic exercise, strengthening, balance and flexibility exercises. Aerobic exercise was prescribed according to performance on the 6-min walk test; exercises included endless band training, cycling, or walking on flat terrain. Strengthening was prescribed according to the scores on the Medical Research Council Muscle sum score, with resistance training by elastic bands, dumbbells, or pulley; the targeted muscles were: deltoid, biceps, triceps, and wrist extensors, iliopsoas, gluteus medius, gluteus maximus, quadriceps, hamstrings, and dorsiflexors; to start rehabilitation, patients performed 1 to 2 sets of 8 to 10 repetitions with 1 min of rest in-between, gradually increasing up to 12 repetitions per set. Then, the number of sets/sessions increased over subsequent weeks. During exercise, patients were encouraged to perform active breathing (abdomino-diaphragmatic/ costobasal). In the first session (s), balance therapy was performed while seated, focusing on ankle and foot proprioception, using a sheet, ball, or cushion; according to progress, exercises gradually transitioned to bipedal stance, with focus on proprioception of the knee and hip joints. Subsequently, exercises were aimed to gait re-education, including unipedal support, semi-tandem, and tandem stance. Muscle flexibility training was targeted to pectorals, latissimus dorsi, trapezius, rhomboids, iliopsoas, hamstrings, and gastrocnemius. Stretch was hold for 30 s to 1 min (until pain-free stretch sensation). Recovery was focused on the continuation of gait or joint movements, decreasing speed for 5 min, with a final rest.

Statistical analysis was performed after Kolmogorov Smirnov test, corresponding to data distribution. The bivariate analysis included “t” test for independent samples, analysis of variance (either for independent measurements or repeated measures) with Duncan test, and Pearson´s coefficient of correlation, or Friedman test, and Spearman coefficient of correlation. According to the results on the bivariate analysis, correlations were evaluated using analysis of covariance, and multivariate analysis of covariance (for repeated measures). The statistical significance was set at 0.05.

### Ethical approval

This study was performed in line with the principles of the Declaration of Helsinki. Approval was granted by the Research and Ethics Committees of Instituto Mexicano del Seguro Social (07–10-2020/2020–785-157), all procedures were performed in accordance with relevant guidelines and regulations.

## Results

### Descriptive and bivariate analyses

#### Characteristics of the participants

The general characteristics by sex are described in Table 1; almost one in three patients had systemic high blood pressure, with a similar frequency of diabetes; while 42% had BMI ≥ 30, almost half reported alcohol consumption, and 27% reported tobacco use. At the three evaluations, the age was related to the FVC and FEV1 (Pearson´s coefficients -0.46 to -0.65, *p* < 0.00001), and to the corrected distance covered during the 6MWT (Pearson´s coefficients 0.36 to 0.55, *p* ≤ 0.005). Additionally, the age was related to the repetitions on the 30-s sit-to-stand-test, to the time to perform the timed-up-and-go-test (Pearson´s coefficients 0.29 to 0.37, *p* ≤ 0.028), and to the vastus medialis thickness (Pearson´s coefficients 0.36 to 0.38, *p* ≤ 0.007); and, in the first evaluation, the age was related to the MRC score (Pearson´s coefficient 0.27, *p* = 0.044). The history of tobacco use was related to more prolonged time of evolution before hospitalization (“t” test, t = 3.058, *p* = 0.003) and to higher total scores and sub-scores on the St George´s Questionnaire, except from the symptoms sub-score (“t” test, t = 2.037 to 3.246, *p* ≤ 0.046). At the three evaluations, the history of diabetes was related to shorter distance covered during the 6MWT, (“t” test, t = 2.014 to 2.576, *p* ≤ 0.048), while the history of high systemic blood pressure was related to higher systolic blood pressure at the first evaluation, and to higher maximum heart rate at the first and third evaluations (“t” test, t = 2.431 and 2.777, *p* ≤ 0.018).

**Table 1:** General characteristics of the 66 patients by sex.

Variable	Women		Men		All	
	(n = 25)		(n = 41)		(n = 66)	
	Mean (S.D.)	Range	Mean (S.D.)	Range	Mean (S.D.)	Range
Years of Age	51.5 (13.1)	28–71	51.1 (15.8)	25–85	51.3 (14.7)	25–85
Days from onset to hospitalization	8.4 (3.8)	1–15	9.6 (3.8)	2.-21	9.2 (3.8)	1–21
Days in Hospital	11.6 (14.0)	1–61	11.2 (10.1)	1–52	11.3 (11.6)	1–61
Days from discharge to rehabilitation referral	125.8 (62.0)	55–353	148.0 (77.6)	42–353	139.9 (72.4)	42–353
Body height (cm)	154.6 (7.4)	141–169	167.6 (5.8)	157–178	162.7 (9.0)	141–178
Body mass at hospital admission (kg)	75.8 (17.3)	58–134	83.2 (14.2)	65–139	80.4 (15.8)	58–139
Body mass at hospital discharge (kg)	74.7 (17.5)	54–131	81.6 (14.0)	64–137	79.0 (15.6)	54–137

	n	%	n	%	n	%
Type 2 diabetes	12	48	13	31	25	37
Systemic High Blood Pressure	10	40	12	29	22	33
Body Mass Index ≥ 30 at hospital admission	13	52	15	36	28	42
Tobacco use	3	12	15	36	18	27
Alcohol consumption	6	24	26	63	32	48

#### Nutrition status

**Figure 1:**
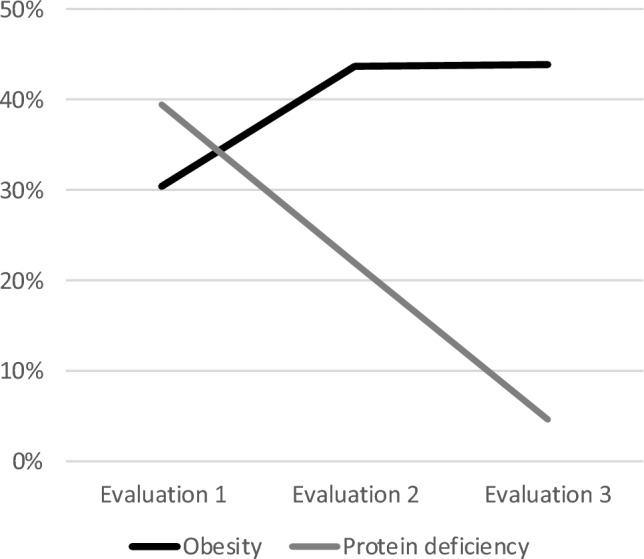
Frequency of obesity and protein deficiency during the follow-up of 6 months.

At the beginning of the study, protein deficiency was evident even among patients with BMI ≥ 25 kg/m^2^ (n = 11), independently from the time elapsed since hospital discharge, or comorbidities. In between the first two evaluations, the frequency of protein deficiency decreased, while the frequency of obesity tended to increase; at the third evaluation, the frequency of protein deficiency decreased further, while the frequency of obesity remained similar (Fig. 1). At the three evaluations, the brachial circumference gradually increased, with no change of the calf circumference (Table 2). During the follow-up, protein deficiency was related to smaller anthropometric measurements (“t” test, t = 2.6318 to 3.991, *p* ≤ 0.011). At the first evaluation, the evidence of protein deficiency was also related to lower albumin concentration at hospital discharge (3.26 S.D. 0.32 *versus* 3.52 S.D. 0.43, “t” test, t = − 2.599, *p* = 0.011). At the second evaluation, protein deficiency was related to smaller muscle thickness of the vastus medialis (1.33 S.D. 0.39 *versus* 1.72 S.D. 0.52; ”t” test, t = − 2.582, *p* = 0.012) and the proportional difference between the two hemidiaphragm thickness at the end of inspiration and expiration (− 0.030 S.D. 0.255 vs. 0.162 S.D. 0.186 for the right hemi-diaphragm; and 0.013 S.D. 0.123 vs. 0.145 S.D. 0.199 for the left hemi-diaphragm; “t” test, t = − 2.256 and − 3.070, *p* ≤ 0.027). At the third evaluation comparisons were not pertinent due to the small number of patients with protein deficiency. According to the nutritional status, at the first and the third evaluations, the absolute distance covered during the 6MWT varied (ANOVA, F = 4.243 and 5.564, *p* ≤ 0.008), with significant differences between patients with adequate nutrition compared to those with undernutrition or obesity (Duncan test, *p* ≤ 0.05).

**Table 2:** Summary of the results at the three evaluations.

Variable	Evaluation 1	Evaluation 2	Evaluation 3	F (p)
	Mean (S.D.)	Mean (S.D.)	Mean (S.D.)	
Body mass	79.2 (15.5)	79.9 (15.9)	80.1 (15.6)	-
Body mass index	29.9 (5.4)	30.2 (5.6)	30.2 (5.6)	-
Brachial circumference	31.7 (4.0)	32.3 (4.1)	32.5 (4.1)	8.171 (0.0004)
Calf circumference	37.2 (3.9)	37.4 (4.3)	37.5 (4.0)	-
Vastus medialis				
Muscle thickness (cm)	1.44 (0.42)	1.64 (0.52)	1.79 (0.53)	16.169 (< 0.00001)
Fascicle length (cm)	4.15 (1.00)	4.09 (0.94)	4.24 (0.96)	-
Shear Wave elastography (m/s)	1.86 (0.57)	2.05 (0.67)	1.76 (0.65)	6.427 (0.002)
Right hemidiaphragm				
Movement	1.92 (0.60)	2.11 (0.65)	2.22 (0.63)	10.379 (0.00006)
Inspiration thickness (cm)	0.32 (0.11)	0.34 (0.15)	0.39 (0.14)	9.084 (0.0002)
Expiration thickness (cm)	0.31 (0.13)	0.28 (0.11)	0.34 (0.12)	5.834 (0.003)
Left hemidiaphragm				
Movement	1.87 (0.64)	2.05 (0.75)	2.26 (0.23)	6.870 (0.001)
Inspiration thickness (cm)	0.32 (0.08)	0.34 (0.12)	0.43 (0.14)	18.889 (< 0.00001)
Expiration thickness (cm)	0.30 (0.09)	0.29 (0.10)	0.37 (0.12)	9.813 (0.0001)
MRC (sum-score)	58.2 (3.2)	58.9 (3.2)	59.8 (9.0)	6.919 (0.001)
30 s Sit to Stand test (repetitions)	10.7 (2.3)	11.4 (2.1)	12.3 (2.1)	18.623 (< 0.00001)
Timed up and go test (sec)	8.7 (1.5)	8.5 (1.4)	8.2 (1.2)	6.518 (0.002)
Six-minute walk test				
- Distance (meters)	451.5 (94.1)	442.1 (85.8)	425.8 (70.4)	5.079 (0.007)
- Distance from predicted (%)	83.9 (18.8)	82.5 (18.1)	79.6(15.7)	4.265 (0.016)
- Initial Oxygen Saturation (%)	92.5 (3.2)	93.4 (2.0)	93.1 (1.9)	3.412 (0.036)
- Minimum Oxygen Saturation (%)	91.7 (3.6)	92.4 (3.1)	92.6 (2.8)	-
- Initial Heart Rate (per minute)	72.6 (9.2)	73.9 (7.4)	71.2 (7.0)	-
- Maximum Heart Rate (per minute)	115.6 (9.6)	108.4 (10.0)	109.4 (9.9)	13.373 (< 0.00001)
- Initial Systolic Blood Pressure (mmHg)	121.6 (10.0)	115.4 (10.8)	117.5 (9.3)	9.145 (0.0001)
- Final Systolic Blood Pressure (mmHg)	133.9 (14.6)	132.2 (14.9)	131.9 (13.5)	-
- Initial Diastolic Blood Pressure (mmHg)	74.3 (7.6)	70.0 (8.3)	71.5 (8.1)	6.613 (0.001)
- Final Diastolic Blood Pressure (mmHg)	81.6 (8.5)	79.2 (9.1)	77.7 (9.1)	4.583 (0.011)
Spirometry				
Forced vital capacity (FVC)	3.6 (1.1)	3.8 (1.0)	3.8 (1.0)	11.833 (0.00002)
1st sec forced expiratory volume (FEV1)	2.9 (0.9)	3.1 (0.8)	3.2 (0.8)	14.995 (< 0.00001)
FEV1/FVC ratio	81.7 (5.5)	82.1 (5.5)	82.9 (5.2)	-
25% Forced expiratory flow	6.2 (2.7)	6.0 (2.6)	5.3 (1.9)	4.290 (0.016)
50% Forced expiratory flow	3.2 (1.3)	3.6 (1.3)	3.4 (1.2)	-
75% Forced expiratory flow	1.3 (0.6)	1.4 (0.5)	1.2 (0.4)	-
Peak expiratory flow	7.5 (2.7)	7.6 (2.6)	6.9 (1.7)	-

St George´s Questionnaire	Median (Q1-Q3)	Median (Q1-Q3)	Median (Q1-Q3)	X^2^ (p)
Symptoms	24.8 (18.5–37.4)	19.2 (9.6–35.3)	15.1 (8.8–23.7)	33.660 (< 0.00001)
Activity	47.5(18.1–59.9)	24.5 (0–49.2)	24.0 (0–45.3)	27.825 (< 0.00001)
Impacts	12.5 (5.4–27.5)	6.3 (1.6–16.2)	4.1 (0–17.4)	34.463 (< 0.00001)
Total	24.5 (13.6–34.7)	13.3 (6.9–30.1)	12.2 (2.5–25.9)	44.440 (< 0.00001)

Comparisons were performed either by analysis of variance or Friedman test according to data distribution, with a significance level of 0.05.

#### Ultrasonography measurements

During the follow-up, the vastus medialis thickness and the shear wave propagation velocity increased, without variation on the fascicle length; simultaneously, the diaphragm thickness and mobility increased (Table 2). No correlation was observed between the vastus medialis measurements and the diaphragm measurements (*p* > 0.05). At the first and second evaluations, the vastus medialis thickness was related to both the body mass and the brachial circumference (Table 3). At the second and third evaluations, the fascicle length of the vastus medialis was related to the body mass; and, at the first and third evaluations, it was related to the calf circumference, with a borderline result at the second evaluation; at the three evaluations, the vastus medialis thickness and fibre length were related to the FVC and FEV1 (Table 3).

**Table 3:** Significant correlations of muscle measurements and scores and sub-scores on the St George´s Questionnaire and the study variables, using Pearson´s coefficient or Spearman coefficient respectively.

Measurement	Evaluation 1	Evaluation 2	Evaluation 3
	Pearson’s r (*p*)	Pearson’s r (*p*)	Pearson’s r (*p*)
Vastus medialis thickness			
Body mass	0.41 (0.004)	0.32 (0.025)	
Brachial circumference	0.34 (0.018)	0.31 (0.031)	
Calf circumference	0.37 (0.009)	–	
Forced vital capacity (FVC)	0.49 (< 0.00001)	0.43 (0.001)	0.35 (0.006)
1st sec forced expiratory volume (FEV1)	0.55 (< 0.00001)	0.43 (< 0.0001)	0.35 (0.007)
Vastus medialis Fascicle length			
Body mass	–	0.35 (0.014)	0.31 (0.029)
Brachial circumference	–	–	–
Calf circumference	0.31 (0.033)	*0.28 (0.051)*	0.37 (0.010)
Forced vital capacity (FVC)	0.37 (0.005)	0.53 (< 0.0001)	0.40 (0.002)
1st sec forced expiratory volume (FEV1)	0.38 (0.004)	0.53 (< 0.0001)	0.35 (0.008)

	Rho (*p*)	Rho (*p*)	Rho (*p*)
Symptoms			
Forced vital capacity	− 0.29(0.016)	− 0.28 (0.024)	–
1st sec forced expiratory volume	− 0.28 (0.022)	− 0.27 (0.028)	–
6MWT Distance (meters)	− 0.35 (0.003)	–	− 0.33 (0.006)
6MWT Distance from predicted	− 0.25 (0.036)	–	− 0.31 (0.010)
6MWT Initial Heart Rate	–	− 0.28 (0.021)	–
6MWT Maximum Heart Rate	–	–	–
Timed up and go test	–	–	–
30 s Sit to Stand test	–	− 0.32 (0.010)	–
Activities			
Forced vital capacity	–	–	–
1st sec forced expiratory volume	–	− 0.26 (0.036)	–
6MWT Distance	− 0.27 (0.023)	− 0.39 (0.001)	− 0.32 (0.008)
6MWT Distance from predicted	− 0.27 (0.025)	− 0.37 (0.002)	–
6MWT Initial Heart Rate		–	− 0.31 (0.011)
6MWT Maximum Heart Rate	0.24 (0.049)	–	− 0.28 (0.020)
Timed up and go test	–	–	0.27 (0.028)
30 s Sit to Stand test	–	–	− 0.31 (0.009)
Impact			
Forced vital capacity	–	–	–
1st sec forced expiratory volume	–	–	–
6MWT Distance	− 0.31 (0.011)	–	–
6MWT Distance from predicted	− 0.32 (0.007)	–	–
6MWT Initial Heart Rate	–	− 0.39 (0.001)	− 0.25 (0.038)
6MWT Maximum Heart Rate	–	–	− 0.40 (0.001)
Timed up and go test	–	–	0.26 (0.31)
30 s Sit to Stand test	–	− 0.32 (0.008)	− 0.37 (0.002)
Total			
Forced vital capacity	–	− 0.24 (0.050)	–
1st sec forced expiratory volume	–	− 0.25 (0.044)	–
6MWT Distance	− 0.34 (0.005)	− 0.27 (0.026)	− 0.31 (0.010)
6MWT Distance from predicted	− 0.33 (0.006)	− 0.26 (0.035)	–
6MWT Initial Heart Rate	–	− 0.31 (0.012)	− 0.29 (0.014)
6MWT Maximum Heart Rate	–	–	− 0.34 (0.004)
Timed up and go test	–	–	0.25 (0.040)
30 s Sit to Stand test	–	− 0.29 (0.016)	− 0.34 (0.005)

#### Muscle strength

The MRC sum-score gradually increased, with an increase on the repetitions during the 30 s sit-to-stand-test (Table 2). Apart from the correlation with the patient age, no other correlations were observed for these measurements.

#### Submaximal exercise capacity and spirometry

During the follow-up, the absolute distance covered during the 6MWT decreased, while the initial oxygen saturation consistently increased, and the maximum heart rate decreased, accompanied by decrease of the systemic blood pressure. During follow-up, the FVC and FEV1 improved, with decrease of the 25% forced expiratory flow. During the first evaluation, the FVC and FEV1 were related to the corrected distance covered during the 6MWT (Pearson´s coefficients − 0.33 and − 0.30, *p* ≤ 0.024), and to the shear wave velocity propagation in the vastus medialis (Pearson´s coefficient 0.29, *p* < 0.028).

#### Mobility

During the follow-up, the time to perform the timed-up-and-go-test gradually decreased (Table 2), with no linear correlation with the other measurements.

#### Health related quality of life

At the first evaluation, the total score and sub-scores were the highest, afterwards all the scores gradually decreased, independently from comorbidities (Table 2). No correlations with the vastus medialis or the diaphragm measurements were observed (Table 3); at the second evaluation, the total score was related to the FVC and FEV1; at the second and third evaluations, correlation was observed with the 30-s sit-to-stand-test; at the three evaluations, correlations were evident with the 6MWT, particularly on the absolute distance covered during the test; just at the third evaluation, correlation was evident with the timed-up-and-go-test. On the sub-scores, at the first and the second evaluations, the symptoms sub-score was related to the FVC and FEV1, to the heart rate and the absolute distance covered in the 6MWT; at the second evaluation, it was related to the 30-s sit-to-stand-test. Through the follow-up, the activity sub-score, was related to the 6MWT, and only at the second evaluation, it was related to spirometry; at the third evaluation it was related to the timed-up-and-go-test, and the 30-s sit-to-stand test. At the second and third evaluations, the impacts sub-score was related to the heart rate in the 6MWT; at the first evaluation, it was related to the distance covered in the 6MWT, and at the second and third evaluations, it was related to the 30-s sit-to-stand-test; also, at the third evaluation, it was related to the timed-up-and-go-test.

#### Self-reported functional status

**Figure 2:**
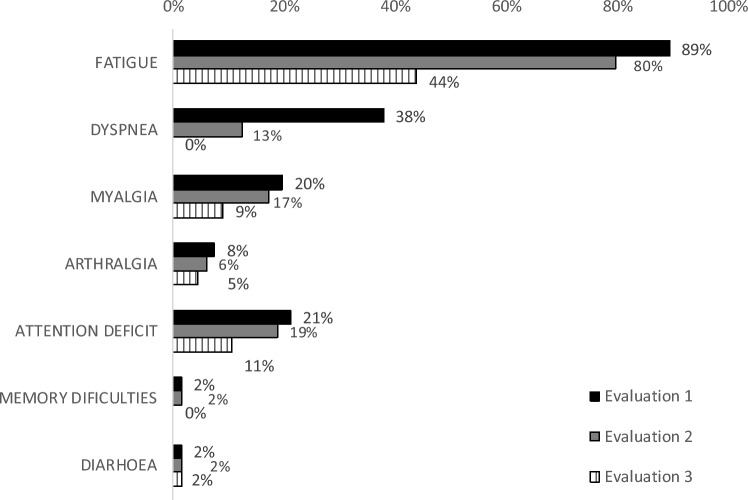
Frequency of the limiting symptoms reported by the patients, at the three evaluations.

At the first evaluation, none of the patients have returned to their daily life activities, 88% reported limitations and 12% were inactive; at the second evaluation, 18% reported to be performing their daily life activities as usual, 80% reported having persistent limitations, and 2% were inactive; at the third evaluation 56% reported to be performing their daily life activities as usual, 42% to have persistent limitations, and 2% were inactive. In the three evaluations, the main persistent symptoms were fatigue and dyspnoea, which gradually decreased, from 89% at the first evaluations to 44% at the third evaluation for fatigue, and from 38 to 0% for dyspnoea (Fig. 2). In the first evaluation, since no patients reported performing their activities as usual, comparisons were performed between those with limitations and those inactive; and for the second and third evaluations, since only one patient remained inactive, comparisons were performed between those reporting recovery and those reporting limitations. At the first evaluation, differences according to functional status were observed on the age of the patients, the FVC and FEV1, and the vastus medialis thickness and fibre length (“t” test, t = − 2.736 to 3.437, *p* ≤ 0.001); at the second evaluation, these differences persisted accompanied by differences on the 30-s sit- to-stand-test, the 6MWT, the timed-up-and-go-test, and the St George´s Questionnaire (“t” test, t = − 2.008 to 3.819, *p* ≤ 0.05); and at the third evaluation, differences were observed on FVC and FEV1 , the 6MWT, the timed-up-and-go-test, and the St George´s Questionnaire (“t” test, t = 2.208 to 2.887, *p* ≤ 0.030). No differences related to the history of diabetes or systemic high blood pressure were observed.

### Multivariate analysis

#### Health related quality of life

Analysis on the St George´s Questionnaire was performed on each evaluation, due to the variability of contributing cofactors. At the first evaluation, contribution to the variance of the total score of the St George´s Questionnaire was mainly from the age of the patients, the FVC, the absolute distance covered during the 6MWT, and the history of tobacco use, which explained circa one third of the variance (Table 4), these relationships were observed almost on all the sub-scores, with no gender influence but a relationship between the symptoms sub-score and the vastus medialis thickness (Table 4). At the second evaluation, the total score was mainly related to the age of the patients, the FVC, and the history of tobacco use, these relationships were observed on all the sub-scores (Table 4). At the third evaluation, the total score was related to the absolute distance and the maximum heart rate during the 6MWT, and to the history of tobacco use, these relationships were variable on each sub-score (Table 4).

**Table 4:** Univariate results on the analysis of covariance on the scores and sub-scores of the St George’s Questionnaire at the three evaluations (6MWT = 6-min-walk-test).

Evaluation/ variables	Symptom sub-score	Activity sub-score	Impact sub-score	Total score
	Univariate F (p)	Univariate F (*p*)	Univariate F (*p*)	Univariate (F, *p*)
Evaluation 1				
*Adjusted R*^*2*^*, (F, p)*	*0.19 (4.852,0.0001)*	*0.27 (6.888,0.0001)*	*0.34 (9.232,* < *0.0001)*	*0.36 (10.148,* < *0.0001)*
Intercept	30.793 (< 0.00001)	40.187 (< 0.00001)	39.851 (< 0.00001)	54.840 (< 0.00001)
Age	5.0211 (0.028)	13.809 (0.0004)	15.682 (0.0002	18.153 (0.00007)
Forced Vital Capacity	8.797 (0.004)	12.561 (0.0007)	10.740 (0.001	15.943 (0.0001)
6MWT Distance	6.669 (0.012)	3.917 (0.052)	6.012 (0.017	7.355 (0.008)
Tobacco smoke	1.5156 (0.223)	9.650 (0.002)	17.821 (0.00008)	14.942 (0.0002)
Evaluation 2				
*Adjusted R*^*2*^*, (F, p)*	*0.23 (5.600, 0.0007)*	*0.21 (5.230, 0.001)*	*0.23 (5.789, 0.0005)*	*0.28 (6.988, 0.0001)*
Intercept	27.967 (< 0.00001)	23.255 (0.00001)	18.950 (0.00005)	28.944 (< 0.00001)
Age	6.402 (0.014)	6.924 (0.010)	6.810 (0.011)	8.910 (0.004)
Forced Vital Capacity	7.535 (0.008)	12.165 (0.0009)	7.891 (0.006)	12.520 (0.0007)
Vastus medialis thickness	4.005 (0.049)	0.008 (0.927)	0.318 (0.574)	0.446 (0.506)
Tobacco use	7.321 (0.008)	11.269 (0.001)	16.231 (0.0001)	16.405 (0.0001)
Evaluation 3				
*Adjusted R*^*2*^*, (F, p)*	*0.13 (4.175, 0.009)*	*0.18 (5.776, 0.001)*	*0.23 (7.465, 0.0002)*	*0.23 (7.559, 0.0002)*
Intercept	11.190 (0.001)	14.578 (0.0003)	17.087 (0.0001)	19.156 (0.0004)
6MWT Distance	6.699 (0.012)	5.972 (0.017)	1.870 (0.176)	5.709 (0.019)
6MWT Max heart rate	1.799 (0.184)	3.973 (0.050)	9.556 (0.002)	6.683 (0.012)
Tobacco use	1.669 (0.201)	3.556 (0.064)	5.928 (0.017)	5.102 (0.027)

Multivariate Analysis of Covariance on the repeated measures of the absolute distance covered during the 6MWT, confirmed the negative contribution of excess body mass to performance at the first and third evaluations (MANCOVA, F = 2.873, *p* = 0.012), with no age effect, but gender effect (MANCOVA, F = 7.314, *p* = 0.009), explaining *circa* 13% of the variance (MANCOVA, adjusted whole model R^2^ 0.14 and 0.12, F = 2.168 and 2.346, *p* ≤ 0.044). 

## Discussion

In this study, patients discharged from hospitalization due to moderate or severe COVID-19 had nutritional deficiencies, with decreased skeletal muscle thickness (vastus medialis and diaphragm). At referral for rehabilitation, self-reported inactivity was related to age, lung function and muscle thickness. Simultaneous nutritional and physical exercise allowed recovery, with improvement of the health-related quality of life and functional status. However, there was a negative influence of the history of tobacco use on the respiratory health related quality of life, and a detrimental effect of the delay to start rehabilitation on functioning. In addition, excess body mass was a hurdle to improve submaximal exercise performance.

These results are consistent with a metanalysis of 35 studies evaluating functional status and/or health related quality of life after acute COVID-19, showing impaired lung function, muscle strength, exercise capacity and persistent fatigue^38^. In addition, this study presented the relevance of an early assessment of skeletal muscle thickness and nutritional status, even after moderate disease, while pondering excess body mass, and support that the quality and quantity of skeletal muscle may have influence on a variety of functions, including respiratory function^15^.

Ultrasound imaging allowed recording of the initial muscle thickness decrease and its recovery, according to reference data^39^. However, no linear relationship was observed between the vastus medialis and the diaphragm measurements, since the thickness increase of the vastus medialis was evident earlier than that of the diaphragm (Table 2), when almost all the inactive patients returned to perform their daily life activities (either with or without limitations). Additionally, just when protein deficiency prevailed, the diaphragm thickness was related to the body mass and the brachial circumference; after recovery, these relationships were not evident anymore. This finding is consistent with studies in the general population showing no linear correlation between diaphragm thickness and body mass index^40^.

Performance on the 30-s sit-to-stand-test and the timed-up-and-go-test improved gradually and was influenced by age, but discordant with the decline on the distance covered in the 6MWT. The 30-s sit-to-stand-test allows assessment of muscle strength and cardiovascular endurance^41^, while performance on the timed-up-and-go-test is strongly related to dynamic balance^29,42^, and both are influenced by age^43,44^. On the other hand, the deficient performance on the distance covered in the 6MWT could be at least partially explained by the complex interaction between the muscle loss and the frequency of excess body mass among the patients. At any age, increased body mass has a negative effect on performance during a 400 m walk test^45^; while, both overweight and underweight has been related to deficient walking speed in the elderly^46^.

Evidence supports that obesity is inversely associated with lung function, independent of physical activity and aerobic fitness^47^; during exercise, adults with obesity may show decreased inspiratory muscle activity because of reduced inspiratory strength and increased ventilatory drive^48^; while intra-abdominal fat may hinder descent of the diaphragm^49^ and reduce the expiratory reserve volume^50^; also, body fat deposition on the thorax wall may also impede expansion and excursion of the rib cage^51^, and it is associated with systemic inflammation^52^. Three months after hospital discharge from acute COVID-19, patients with overweight or obesity may also have weight fluctuations with abdominal adiposity^53^. Six months after hospital discharge, adults with obesity and long COVID-19 may display impaired oxygenation at peak exercise with decreased ventilatory reserve, with decreased cardiac function^54^. Conversely, malnutrition is among the persistent sequelae after hospitalization due to COVID-19^55^. Larger studies are needed to assess the contribution of excess body mass on the recovery of patients after COVID-19.

The first limitation of this study is the lack of information on sarcopenia before acute COVID-19. The second limitation is the absence of a control group of patients discharged from hospitalization for other reasons, due to the limited access to hospitalization during the study period. However, the selection of participants allowed exclusion of health conditions that could obscure the results; and the repeated measures design with standardized assessments (including imaging) supplied an approach to individual changes through time. The results underline the relevance of both early nutritional advise and rehabilitation referral to overcome inactivity after hospital discharge due to COVId-19. Future studies may assess reproducibility in a variety of settings, while further assessing contributing cofactors for patient recovery.

After hospital discharge due to moderate to severe COVID-19, nutrition deficiency and decreased muscle thickness may persist, independently from the time elapsed after the acute disease, or comorbidities, or the evidence of excess body mass. Rehabilitation exercises and nutrition advise may reverse these conditions, with an impact on the overall performance of the patients. Early diagnosis of protein deficiency and decline of skeletal muscle mass could be relevant to promote rehabilitation after hospitalization due to moderate to severe COVID-19, while pondering a negative effect from excess body mass.

## Data Availability

The datasets are available from the corresponding author upon reasonable request. All the data are presented within the article.
